# Occurrence of portal venous gas following routine endoscopy: a case report

**DOI:** 10.1515/med-2026-1474

**Published:** 2026-06-24

**Authors:** Liping Yang, Chao Lu

**Affiliations:** Department of Gastroenterology, The First Affiliated Hospital, Zhejiang University School of Medicine, Hangzhou, China; Department of Nursing, The First Affiliated Hospital, Zhejiang University School of Medicine, Hangzhou, China

**Keywords:** portal venous gas, endoscopy, obstruction

## Abstract

**Objectives:**

Portal venous gas (PVG) is commonly associated with severe gastrointestinal disease, whereas benign causes related to endoscopic procedures are uncommon. We report an unusual mechanism of PVG occurring in the setting of chronic gastric outlet obstruction after carbon dioxide (CO_2_)-insufflated upper gastrointestinal endoscopy.

**Case presentation:**

A 25-year-old Asian woman presented with recurrent vomiting. She had undergone surgery for duodenal perforation six months earlier and was subsequently lost to follow-up. Her symptoms recently worsened, with significant abdominal distension and vomiting for 3 weeks. A computed tomography (CT) revealed marked gastric dilatation and fibrotic duodenal bulb stenosis. After gastric decompression, CO_2_-insufflated gastroscopy was performed while she remained conscious. Severe gastric distension, retained contents, and limited procedural tolerance and cooperation limited visualization, and an ultrathin gastroscope could not pass <1 mm stenosis. The procedure was terminated without any immediate complications. Later that night, she developed abdominal pain. CT demonstrated extensive PVG and submucosal gastric wall air, with normal inflammatory markers and no signs of perforation. Conservative treatment with continued decompression and acid suppression led to rapid clinical improvement, and repeat CT two days later showed complete resolution of PVG. Endoscopy used standard low-flow CO_2_ insufflation, total procedure duration was approximately 20 min, and intermittent suctioning was performed for content removal.

**Conclusions:**

In the setting of chronic gastric outlet obstruction and prolonged gastric distension, impaired mucosal integrity together with increased intragastric pressure may predispose to abnormal CO_2_ migration into the portal venous system during endoscopy. Possible partial-thickness or submucosal injury during manipulation may further facilitate portal venous gas formation. Recognition of etiologies of PVG that do not require surgery is essential, as many stable patients can be managed conservatively, preventing unnecessary operative intervention.

## Background

Portal venous gas (PVG) is traditionally regarded as a radiologic sign associated with severe or life-threatening conditions, including bowel ischemia, necrotizing enterocolitis, or gastrointestinal perforation [1], 2]. Historically, hepatic portal venous gas (HPVG) carried a very high mortality rate and frequently indicated transmural bowel necrosis requiring urgent surgery [3]. However, benign causes of PVG have increasingly been recognized, particularly in association with endoscopic procedures and mucosal injury [4], 5]. Endoscopic CO_2_ insufflation is generally considered safe due to its rapid absorption and low risk of embolic complications [6]. Reports of PVG resulting solely from CO_2_ diffusion during upper gastrointestinal endoscopy remain rare, especially in the absence of perforation or systemic inflammation [4], 7].

This case highlights a novel mechanism of endoscopy-associated PVG: chronic fibrotic gastric outlet obstruction, isolated CO_2_ insufflation without dilation/biopsy/PEG, no perforation/ischemia, and rapid conservative resolution – distinct from all prior reports.

## Case presentation

A 25-year-old Asian woman presented with a one-year history of recurrent postprandial vomiting. Six months earlier, she had undergone emergency surgery for a duodenal perforation but failed to follow up post-operatively. She continued to experience intermittent vomiting due to non-compliant with medications for presumed ulcer-related symptoms. On admission, the patient was hemodynamically stable. Physical examination revealed mild upper abdominal distension with epigastric tenderness but no guarding, rebound tenderness, or peritoneal signs. She had no remarkable family history of gastrointestinal disease and no significant psychosocial or substance-use history.

**Figure 1:**
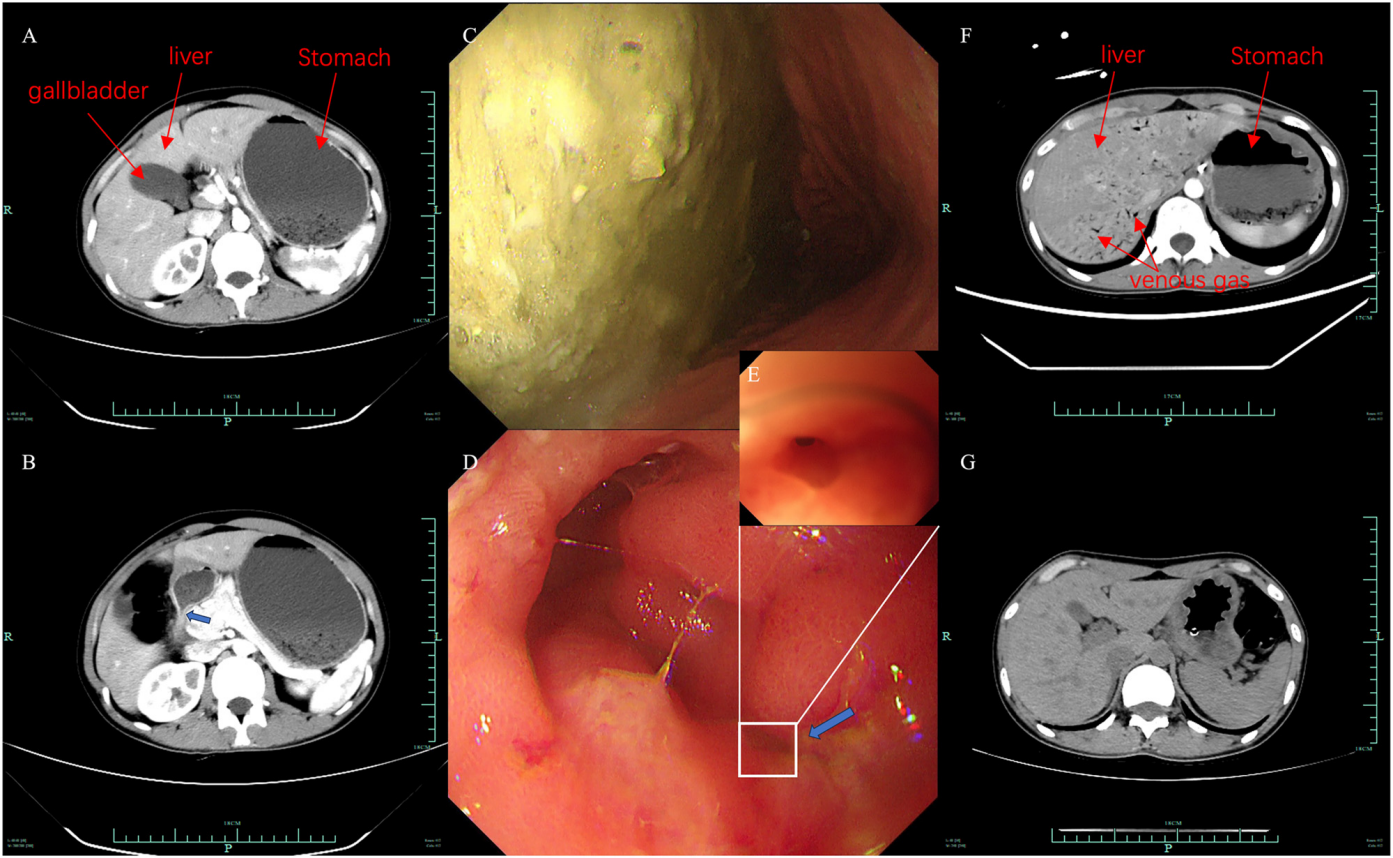
Imaging and endoscopic features of duodenal bulb stenosis with secondary gastric dilatation and transient portal venous gas. (A) Contrast-enhanced CT shows massive gastric dilatation. (B) CT reveals a tight, fibrotic stenosis at the duodenal bulb. (C) Gastroscopy demonstrates a markedly distended stomach filled with retained chyme. (D) The duodenal bulb appears distorted on endoscopy. The area indicated by the blue arrow is the narrow part. (E) A critically narrowed lumen (<1 mm) prevents passage of an ultrathin gastroscope. (F) Emergency CT shows extensive portal venous gas and submucosal air in the gastric wall without free air. (G) Follow-up CT two days later shows complete resolution of portal venous gas with only mild gastric wall edema.

One week prior to admission, her vomiting worsened significantly. Contrast-enhanced CT (on presentation) revealed marked gastric dilatation and a tight, fibrotic stenosis at the duodenal bulb (Figure 1A and B). A nasogastric tube was placed for decompression, and gastroscopy was scheduled. At presentation, CT was performed on hospital day 1, gastroscopy on day 2, and portal venous gas was detected by CT several hours after the procedure. Follow-up CT was obtained two days later showing complete resolution. Endoscopy was performed with CO_2_ insufflation. To reduce aspiration risk, the procedure was performed with the patient conscious; however, she was poorly cooperative. The stomach was massively distended and contained large amounts of retained chyme (Figure 1C). The duodenal bulb was distorted but showed no active ulceration or tumorous changes on endoscopic inspection. Contrast-enhanced CT revealed no evidence of gastric or duodenal mass lesions, and the stenosis appeared fibrotic rather than neoplastic in morphology. Biopsy was not feasible because the lumen was less than 1 mm and could not be traversed even with an ultrathin endoscope (Figure 1D and E). The procedure was terminated without immediate complications. Later that night, the patient developed acute abdominal pain. Emergency CT demonstrated extensive portal venous gas and prominent submucosal air within the gastric wall, without signs of free air or peritonitis (Figure 1F). Laboratory tests revealed normal inflammatory markers. Conservative treatment – including continued gastric decompression and proton-pump inhibitor therapy – was initiated. Her symptoms improved rapidly, and repeat CT after two days showed complete disappearance of PVG and only mild gastric wall edema (Figure 1G). Pathological reports from the previous duodenal perforation surgery were unavailable because the operation had been performed at another institution and no tissue diagnosis had been documented. After clinical stabilization, repeat endoscopy under sedation was recommended; however, the patient declined further invasive evaluation during this admission and opted for elective outpatient management. Long-term follow-up imaging was therefore not available. upper gastrointestinal contrast series was not performed during this admission because cross-sectional CT had already demonstrated the site of obstruction and the patient declined additional invasive evaluation. Overall imaging examination timeline: baseline CT (day 1) → gastroscopy (day 2) → post-procedure CT (evening day 2) → follow-up CT (day 4). PVG was confined to peripheral portal branches, gastric emphysema (submucosal air) was present, and no extra-gastric free air or ischemic signs were detected.

**Research ethics**: The study was approved by the Clinical Research Ethics Committee of the First Affiliated Hospital, Zhejiang University School of Medicine, with a waiver of informed consent.

**Informed consent**: Not applicable.

## Discussion and conclusions

PVG is traditionally considered a marker of life-threatening gastrointestinal pathology. However, accumulating evidence indicates that not all PVG represents an acute surgical emergency [2], 8]. Benign PVG has been described in association with endoscopic procedures, excessive mucosal insufflation, inflammatory conditions, and increased intraluminal pressure. In the present case, several predisposing factors likely contributed to the development of CO_2_-related PVG. Causality is not definitively established; PVG arose from multifactorial interactions: pre-existing gastric distension, mucosal fragility, prior duodenal perforation, and procedural CO_2_ delivery. Although endoscopic CO_2_ insufflation likely acted as a triggering factor, the development of HPVG in this patient was probably multifactorial rather than attributable to a single cause. Red flags for high-risk PVG include hemodynamic instability, elevated lactate, leukocytosis, peritonitis, and free intraperitoneal air. This patient had none of these red flags, confirming a benign etiology. Mechanistically, chronic gastric outlet obstruction leads to prolonged gastric distension, which causes mucosal disruption and exposes submucosal veins; under elevated intragastric pressure, CO_2_ can dissect into the portal venous circulation. Prior duodenal perforation further weakened gastric mucosal integrity. Chronic gastric outlet obstruction with longstanding distension may compromise mucosal barrier integrity and expose the submucosal venous plexus, facilitating intramural and portal gas entry under elevated intraluminal pressure [9]. Importantly, no radiologic or endoscopic evidence of gastric or duodenal tumors or active ulceration was identified during this admission, and inflammatory markers remained normal, arguing against ischemic or malignant etiologies as the primary cause [10].

The absence of peritoneal signs, stable vital signs, and normal inflammatory markers strongly supported a benign etiology rather than bowel ischemia or perforation. Conservative management rationale: normal inflammatory markers, no peritoneal signs, stable hemodynamics, no free air or ischemic signs – surgery was not indicated in this patient. Conservative management is appropriate in such cases and typically leads to rapid resolution, as demonstrated in this patient. Key limitations of this report include: lack of histopathological confirmation from the prior duodenal perforation surgery; inability to obtain endoscopic biopsy due to severe duodenal stenosis; and no post-sedation endoscopy or long-term follow-up imaging. Therefore, occult underlying pathology cannot be fully excluded.

This case highlights a rare but important mechanism of HPVG following gastroscopy in patients with chronic gastric outlet obstruction. Endoscopists should apply gentle insufflation, avoid excessive intragastric pressure, and abort the procedure when visualization is limited in patients with severe gastric distension or a history of perforation.
